# Streamlined Fabrication of Hybrid Lipid Bilayer Membranes on Titanium Oxide Surfaces: A Comparison of One- and Two-Tail SAM Molecules

**DOI:** 10.3390/nano12071153

**Published:** 2022-03-30

**Authors:** Tun Naw Sut, Sue Woon Tan, Won-Yong Jeon, Bo Kyeong Yoon, Nam-Joon Cho, Joshua A. Jackman

**Affiliations:** 1 School of Chemical Engineering and Translational Nanobioscience Research Center, Sungkyunkwan University, Suwon 16419, Korea; suttunnaw@skku.edu (T.N.S.); suewoon4695@gmail.com (S.W.T.); powerwy@skku.edu (W.-Y.J.); 2 School of Healthcare and Biomedical Engineering, Chonnam National University, Yeosu 59626, Korea; 3 School of Materials Science and Engineering, Nanyang Technological University, 50 Nanyang Drive, Singapore 637553, Singapore

**Keywords:** hybrid lipid bilayer, self-assembled monolayer, titanium oxide, surface functionalization

## Abstract

There is broad interest in fabricating cell-membrane-mimicking, hybrid lipid bilayer (HLB) coatings on titanium oxide surfaces for medical implant and drug delivery applications. However, existing fabrication strategies are complex, and there is an outstanding need to develop a streamlined method that can be performed quickly at room temperature. Towards this goal, herein, we characterized the room-temperature deposition kinetics and adlayer properties of one- and two-tail phosphonic acid-functionalized molecules on titanium oxide surfaces in various solvent systems and identified optimal conditions to prepare self-assembled monolayers (SAMs), upon which HLBs could be formed in select cases. Among the molecular candidates, we identified a two-tail molecule that formed a rigidly attached SAM to enable HLB fabrication via vesicle fusion for membrane-based biosensing applications. By contrast, vesicles adsorbed but did not rupture on SAMs composed of one-tail molecules. Our findings support that two-tail phosphonic acid SAMs offer superior capabilities for rapid HLB coating fabrication at room temperature, and these streamlined capabilities could be useful to prepare durable lipid bilayer coatings on titanium-based materials.

## 1. Introduction

The fabrication of durable lipid bilayer coatings is broadly relevant to the design of various biointerfaces such as biosensing platforms as well as to medical implant surfaces [1]. Such coatings can endow inorganic material surfaces with biocompatibility, antifouling, and biomimetic functions by recapitulating key structural properties of cell-membrane-mimicking lipid bilayers [2,3]. Currently, there are two main classes of supported lipid bilayer (SLB) coatings that are distinguished by how the lower leaflet of the SLB is coupled to the surface, either noncovalently or covalently. In the former case, the conventional fabrication method is the adsorption and spontaneous rupture of lipid vesicles on a hydrophilic surface, whereby the lipid molecules reassemble to form a conformal SLB coating [4,5,6], while other methods such as solvent-assisted lipid bilayer (SALB) [7,8,9] and bicelle deposition [10,11] are also possible.

The latter case is sometimes referred to as a hybrid lipid bilayer (HLB) and is understood to improve the ruggedness of lipid bilayer attachment [12,13,14,15]. In this case, HLB fabrication typically involves two main steps: (1) a hydrophobic self-assembled monolayer (SAM) is first formed on the material surface (usually gold) by utilizing amphipathic molecules with one side bearing a functional group that can attach covalently to the surface and the other side consisting of a hydrocarbon chain (or so-called “tail”; in general, such molecules have one tail while two-tail molecules are also possible) [16,17,18,19]. This SAM layer constitutes the lower leaflet of the eventual HLB; and (2) addition of lipid vesicles that can fuse with the hydrophobic SAM to form the upper leaflet of the HLB, resulting in its completion [20,21]. For step (2), it is alternatively possible to conduct a rapid solvent-exchange step whereby lipids in a water-miscible organic solvent are incubated with the SAM-coated surface, followed by solvent-exchange to an aqueous media [7]. An important distinction between the noncovalent and covalent cases is that the lower and upper leaflets are typically similar in the former case, while the specific compositional properties of the two leaflets can be tuned controllably in the latter case.

Among different materials options, titanium-based surfaces are one of the most important for developing SLB coatings because they are widely used as implant materials [22,23], and noncovalent adsorption of conventionally used zwitterionic lipid vesicles is typically insufficient to form SLBs on titanium-based surfaces [24]. Rather, vesicle adsorption on titanium oxide typically results in a single layer of intact vesicles. To overcome this limitation, some surface functionalization strategies have recently been reported to facilitate the formation of SLB coatings on titanium-based surfaces. For example, Valiūnienė et al. reported the silanization of a titanium film to obtain a hydrophobic silanized SAM, which then acts as a cushion for phospholipids to form an HLB via vesicle fusion [25]. Sabirovas et al. further investigated the effects of the organic solvent used for surface cleaning and of heating the surface prior to silanization on the resulting SAM properties, and demonstrated HLB formation on the silanized SAM with phospholipids containing 40 mol% cholesterol along with reusability of the silanized SAM [26]. It has also been shown that a silanized SAM could be formed with a mixture of long and short silanes to create aqueous reservoirs below the short silanes, which can potentially be useful for protein reconstitution into the HLB [27]. Within this context, tethered lipid bilayers [28]—a specific type of HLB—are also noteworthy, and the use of silanization to directly tether phospholipids to titanium can result in a silane-tethered phospholipid SAM, onto which another layer of phospholipid can assemble to form an HLB [29]. Collectively, these studies have shown the possibility of HLB formation on titanium; however, there are also challenges such as the need for additional steps to prepare the surface (e.g., vapor deposition [25] or mechanical polishing [26,27]), extensive use of organic solvents during silanization, high temperature, and long fabrication time, all of which can limit HLB practicality in terms of fabrication and application.

Aside from silanization-based approaches, other recent findings demonstrate that in situ formation of an HLB coating on titanium oxide nanoparticles can be achieved rapidly in aqueous buffer conditions through simple mixing with lipid vesicles composed of negatively charged inverse-phosphocholine (CP) lipids that bear terminal phosphate groups [30,31]. It has also been shown that CP lipid vesicles can adsorb and rupture on planar titanium oxide surfaces to form an HLB coating as well [32]. In spite of strong electrostatic repulsion between the negatively charged vesicles and titanium oxide surface, the contacting CP lipid vesicles anchor to the titanium oxide surface through coordinate bonding, undergo extensive deformation if there is a sufficient number of binding contact sites per attached vesicle, and proceed to fuse and rupture once a critical surface coverage of attached vesicles is reached [32]. Notably, the CP lipid essentially functions as a two-tail SAM molecule, and the upper leaflet can be removed with ethanol washing [32], allowing it to potentially serve as a template for HLB fabrication with adjustable upper leaflet compositions. On the other hand, numerous single-tail, phosphonic acid molecules have been utilized to form monolayers on titanium oxide in other surface functionalization contexts [33,34,35,36] but remain unexplored for HLB coating fabrication. Hence, the comparative investigation of one- and two-tail phosphonic acid-functionalized molecules to form HLB coatings on titanium oxide surfaces could help define the utility scope, refine mechanistic understanding, and guide the development of practically useful HLB surface functionalization strategies for titanium oxide surfaces.

Herein, we characterized the deposition kinetics and adlayer properties of one- and two-tail phosphonic acid-functionalized molecules on titanium oxide surfaces that were directly prepared in aqueous and organic solvent conditions and tracked subsequent interactions with biotinylated lipid vesicles. In some, but not all cases, the phosphonic acid-functionalized molecules formed rigid adlayers, which was a necessary step to support vesicle adsorption and/or rupture. In successful cases, the resulting HLB coatings enabled selective detection of target protein antigens and antibodies in multistep interactions. Our findings support that two-tail phosphonic acid SAMs offer superior capabilities for HLB coating fabrication in near-physiological pH conditions and also provide clues for how the molecular properties of one- and two-tail phosphonic acid-functionalized molecules might be tuned to guide biomimetic surface functionalization of lipid bilayer coatings on titanium oxide surfaces.

## 2. Materials and Methods

### 2.1. Reagents

1,2-dioleoyl-*sn*-glycero-3-phosphocholine (DOPC, product no.: 850375, >99% purity), 1,2-dioleoyl-*sn*-glycero-3-phosphoethanolamine-N-(biotinyl) (sodium salt) (Biotinyl PE, product no.: 870282, >99% purity), and 2-((2,3-bis(oleoyloxy)propyl)dimethylammonio)ethyl hydrogen phosphate (DOCP, product no.: 850311, >99% purity) lipids were supplied in chloroform stock solution from Avanti Polar Lipids (Alabaster, AL, USA). Octadecylphosphonic acid (ODPA, product no.: 715166, 97% purity), dodecylphosphonic acid (DPA, product no.: 795755, 97% purity), bovine serum albumin (BSA, product no.: A7030, ≥98% purity), and other reagents were purchased from Sigma Aldrich (St. Louis, MO, USA). Tris buffer solution consisting of 10 mM Tris and 150 mM NaCl was prepared using Milli-Q-treated deionized water (MilliporeSigma, Burlington, MA, USA), and the solution pH was adjusted to 7.5.

### 2.2. Self-Assembled Monolayer (SAM) Preparation

DOCP samples were prepared by drying the required amount of DOCP lipid in chloroform with a gentle stream of nitrogen gas and then hydrating the sample in ethanol or Tris buffer. ODPA and DPA samples were supplied in the lyophilized state and were prepared by weighing the required amount, followed by dissolving the sample in ethanol or Tris buffer. The concentration of SAM molecules was fixed at 0.5 mM.

### 2.3. Vesicle Preparation

Phospholipid vesicles containing DOPC/Biotinyl PE lipids at the molar ratio of 100/0 or 97/3 were prepared by the extrusion method [37]. Briefly, the phospholipids were mixed in chloroform to the desired molar ratio. Then, the mixture was dried with a gentle stream of nitrogen gas until a dry lipid film was formed along the sidewall of a glass vial before placing it in a desiccator overnight to remove residual chloroform. After hydrating the lipid film with Tris buffer solution and vortexing for 3 min, the vesicles were extruded through polycarbonate membranes with 50 nm diameter pores by using a Mini-Extruder (Avanti Polar Lipids, Alabaster, AL, USA). The final stock concentration of lipid vesicles was 5 mg/mL. Dilution was performed immediately before vesicle addition during each experiment.

### 2.4. Protein Biotinylation

BSA protein was functionalized with biotin moieties by using an EZ-Link Sulfo-NHS-LC-Biotinylation Kit (Thermo Scientific, Waltham, MA, USA). The manufacturer’s protocol was followed, whereby the BSA concentration was 5 mg/mL in phosphate-buffered saline (PBS) solution and the biotin-to-BSA molar ratio was 20:1. After reaction, free biotin was removed by using a spin desalting column. The stock concentration of biotinylated BSA was 90 µM and was diluted to 2 µM in Tris buffer before the experiment.

### 2.5. Quartz Crystal Microbalance-Dissipation (QCM-D)

A Q-Sense E4 instrument (Biolin Scientific AB, Stockholm, Sweden) was used for all QCM-D experiments involving deposition kinetics and interaction processes. Liquid samples were injected into the measurement chamber by using a Reglo Digital MS-4/6 peristaltic pump (Ismatec, Glattsburg, Switzerland), and the flow rate was 50 µL/min. TiO_2_-coated QCM-D sensor chips (product no.: QSX310, Biolin Scientific AB, Gothenburg, Sweden) were used for experiments, and the main TiO_2_ phase on the sensor surface was anatase along with titanium [38,39]. Prior to the experiment, the sensor chips were sequentially rinsed with 1% (*w/v*) aqueous sodium dodecyl sulfate, deionized water, and ethanol, and then dried under a gentle stream of nitrogen gas. The rinsed chips were then treated with oxygen plasma for 2 min in a CUTE-1MPR oxygen plasma chamber (Femto Science Inc., Hwaseong, Korea) before being mounted in the QCM-D measurement chambers. In each experiment, a stable baseline signal was first established in aqueous buffer and the reported data were collected at the 5th odd overtone. In applicable cases, the Sauerbrey equation [40] was applied to calculate the surface mass density of adsorbed molecules, and the mass sensitivity constant used in the calculations was 17.7 ng/cm^2^ per 1 Hz shift.

## 3. Results and Discussion

### 3.1. Experimental Strategy

**Figure 1:**
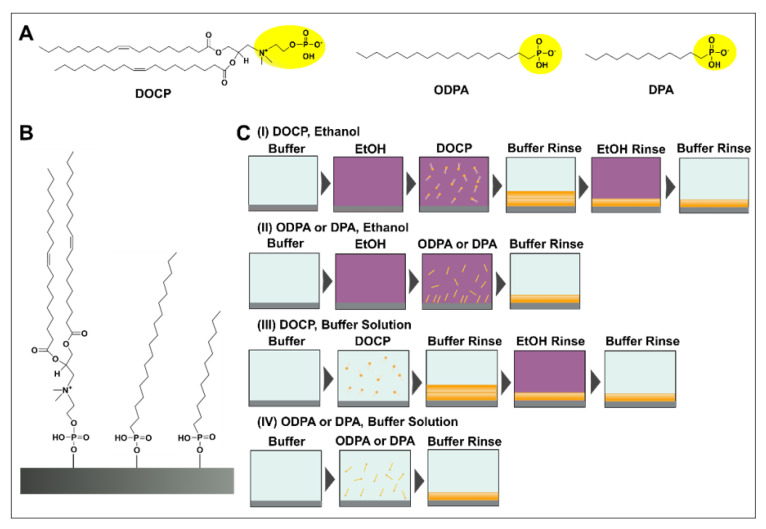
(**A**) Molecular structures of the one- and two-tail amphipathic molecules functionalized with phosphate groups. (**B**) Proposed schematic of DOCP, ODPA, and DPA (from the left) molecules bonding to a TiO_2_ surface based on coordination chemistry. (**C**) Overview of experimental strategy for coating phosphate-functionalized molecules onto a TiO_2_ surface based on deposition in (**I**,**II**) ethanol or (**III**,**IV**) buffer solution.

The chemical structures of the coating molecules used in this study, namely bis(((oleoyloxy)propyl)dimethylammonio)ethyl hydrogen phosphate (DOCP), octadecylphosphonic acid (ODPA), and dodecylphosphonic acid (DPA), are presented in Figure 1A. DOCP is a phospholipid and has two, 18-carbon long hydrocarbon tails (bi-tail) with one degree of unsaturation each, whereas ODPA has a single, 18-carbon long hydrocarbon tail (mono-tail) that is fully saturated. Likewise, DPA has a 12-carbon long hydrocarbon tail (mono-tail) that is also fully saturated. All three amphipathic molecules have a phosphate-functionalized headgroup that can chemically interact with TiO_2_ surfaces via P-O-Ti coordinate bond formation [41] and were hence explored as prospective SAM candidates (Figure 1B). Of note, the phosphate group on the DOCP headgroup has primary and secondary p*K*_a_ values of 2.83 and 7.92, respectively, as determined by potentiometric measurements. For ODPA, the measured primary and secondary p*K*_a_ values of its phosphate group are 1.80 and 7.75, respectively, and the corresponding values for the phosphate group in DPA are 1.80 and 7.57.

Figure 1C outlines the experimental strategy for depositing phosphate-functionalized molecules on a TiO_2_ surface. While many protocols reported in the literature utilize long deposition times of one day or longer [42,43,44], we focused on streamlined deposition in 2 h or less, which is more suitable for HLB fabrication. Furthermore, most reported protocols utilize high temperature (50 °C and above) for surface treatment during SAM formation, while we focused on identifying suitable, room temperature conditions. We tested molecular deposition in ethanol and buffer conditions, and experiments were conducted in a flow-through microfluidic chamber. The extent of deposition was measured by the quartz crystal microbalance-dissipation (QCM-D) technique [45], which tracks resonance frequency (Δf) and energy dissipation (ΔD) signals that are related to the mass and viscoelastic properties of the adlayer, respectively. In all experiments, a buffer baseline was recorded, and all reported Δf and ΔD shift values are relative to the buffer baseline. For ethanol deposition, the buffer baseline was followed by solvent-exchange to ethanol, compound addition in ethanol, and buffer rinsing (see Figure 1C, panels I and II). In the DOCP case, an additional sequence of ethanol and buffer washing steps was then performed to remove the upper leaflet of attached DOCP molecules in applicable cases of SAM formation. For buffer deposition, the buffer baseline was followed by compound addition in buffer and buffer rinsing (see Figure 1C, panels III and IV). Again, in the DOCP case, an additional sequence of ethanol and buffer washing steps was performed to remove the upper lipid leaflet in applicable cases.

### 3.2. Characterization of Molecular Deposition Processes

**Figure 2:**
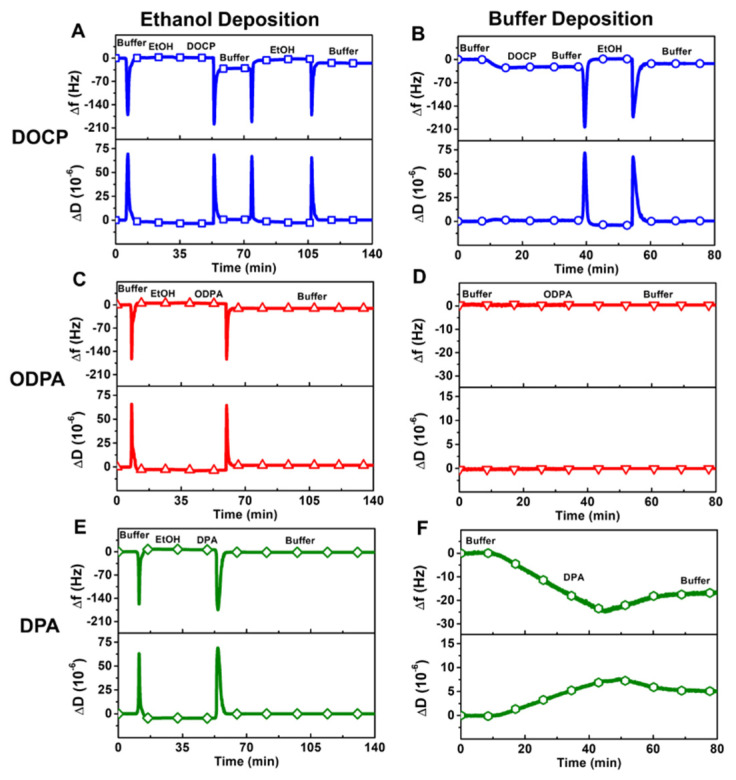
Real-time QCM-D tracking of molecular deposition processes on TiO_2_ surface in different solvent systems. Time-resolved ∆f and ∆D signals are reported for DOCP attachment in (**A**) ethanol and (**B**) buffer. Corresponding data for (**C**,**D**) ODPA and (**E**,**F**) DPA. In all cases, a buffer baseline step was initially established, and subsequent steps labeled as EtOH and buffer refer to ethanol and buffer washing steps, respectively.

Figure 2 presents the time-resolved QCM-D ∆f and ∆D shifts for DOCP, ODPA, and DPA deposition in ethanol and buffer solution, and each molecule exhibited distinct deposition behaviors. In general, larger ∆f shift decreases reflect greater mass uptake while larger ∆D shift increase are related to more viscoelastic film character arising from more hydrodynamically coupled solvent and/or less ordered molecular arrangements.

#### 3.2.1. DOCP

For DOCP in ethanol, there was initially minimal lipid adsorption in ethanol, however, an SLB was then rapidly formed upon solvent-exchange from the lipid-containing ethanol solution to neat buffer solution, yielding ∆f and ∆D shifts of −25.4 ± 3.5 Hz and 0.4 ± 0.1 × 10^−6^, respectively, relative to the initial buffer baseline (Figure 2A). The ∆f and ∆D shifts are indicative of a complete SLB forming on the TiO_2_ surface [45], supporting that DOCP lipids can spontaneously self-assemble from an ethanol dispersion into an SLB upon a solvent-exchange-type exchange process due to the interplay of coordinate bonding and hydrophobic forces. Based on the intended fabrication goal, we then performed an additional ethanol washing step to remove the upper lipid leaflet, followed by solvent exchange back to neat buffer solution. The corresponding final ∆f and ∆D shifts were −12.2 ± 1.1 Hz and 0.1 ± 0.1 × 10^−6^, respectively, relative to the initial buffer baseline, and were consistent with an attached SAM remaining on the TiO_2_ surface. This conclusion was indicated by (1) attached lipid remaining bound even after ethanol washing and (2) the surface mass density of the attached lipid, which was computed to be around 216 ng/cm^2^ based on the Sauerbrey relationship and was around half the value corresponding to a complete SLB.

On the other hand, for DOCP in buffer solution, the lipid-containing buffer was initially added to the TiO_2_ surface, and one-step adsorption kinetics were observed (Figure 2B). The resulting ∆f and ∆D shifts were −21.0 ± 1.2 Hz and 0.4 ± 0.5 × 10^−6^, respectively, after a buffer rinsing step and were consistent with the typical range for SLB formation. As such, an ethanol washing step was then performed to remove the upper lipid leaflet, followed by solvent exchange back to neat buffer solution. The corresponding final ∆f and ∆D shifts were −11.9 ± 2.0 Hz and 0.1 ± 0.1 × 10^−6^, respectively, relative to the initial buffer baseline, and these values indicated the formation of an attached SAM on the TiO_2_ surface. Hence, DOCP lipids dispersed in ethanol or buffer solution could readily form an SAM on the TiO_2_ surface at room temperature in under 2 h and with no sample preparation besides simple dispersion (see also Figure S1 from the Supplementary Materials). The low ∆D shifts of the adsorbed DOCP lipids further support that the fabricated SAMs were rigidly attached to the TiO_2_ surface.

#### 3.2.2. ODPA

For ODPA in ethanol, there was again minimal molecular adsorption during the initial ethanol deposition step (Figure 2C). However, upon solvent-exchange back to neat buffer solution, the final ∆f and ∆D shifts were around −11.9 ± 2.1 Hz and 2.5 ± 1.1 × 10^−6^, respectively, which indicated SAM formation. Compared to that of the DOCP SAM, the ∆D shift of the ODPA SAM was larger and possibly related to a higher chain tilt angle in the ODPA case. For example, it has been previously reported that ODPA SAMs have chain tilt angles around 30° [46,47]. In marked contrast, for ODPA in buffer solution, there was negligible molecular adsorption, and the corresponding ∆f and ∆D shifts were around 0.1 ± 0.8 Hz and 0.0 ± 0.2 × 10^−6^, respectively (Figure 2D). This lack of adsorption is likely attributable to the relatively poor solubility of long-chain ODPA in aqueous buffer, while the data support that ethanol is a suitable deposition medium to fabricate an ODPA SAM.

#### 3.2.3. DPA

For DPA in ethanol, there was minimal lipid adsorption during the initial ethanol deposition step and, in this case, there was still negligible adsorption after the solvent-exchange to buffer solution (Figure 2E). The corresponding ∆f and ∆D shifts were −0.5 ± 1.7 Hz and ~0 × 10^−^^6^, respectively, which indicated that negligible adsorption occurred. By contrast, for DPA in buffer solution, there were markedly larger ∆f and ∆D shifts around −25 Hz and 8 × 10^−6^, respectively, during the initial deposition step (Figure 2F). After a buffer washing step, the resulting ∆f and ∆D shifts were −18.1 ± 0.9 Hz and 5.1 ± 1.0 × 10^−6^, respectively, which were out of the monolayer range [6,48] and hence supported that a DPA SAM did not form.

In addition, the large ∆D shifts further indicated that the adsorbed layer was not rigid. Compared to DOCP and ODPA, DPA has a shorter chain length and a chain tilt angle of around 45° [47], which likely results in a less dense and more disordered adsorbed layer due to weaker interchain van der Waals interactions between DPA molecules. Moreover, since the DPA molecules have relatively low solubility in buffer, the adsorption of DPA aggregates may be another reason giving rise to the large ∆D shifts.

**Figure 3:**
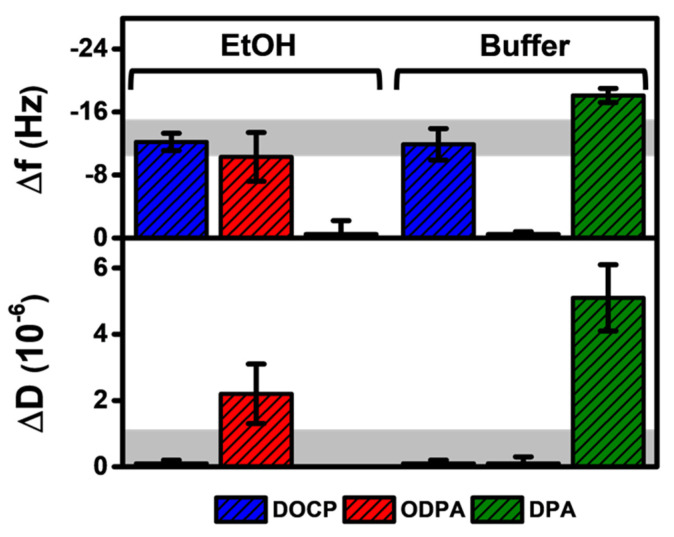
Summary of QCM-D measurement responses corresponding to DOCP, ODPA, and DPA attachment on TiO_2_ surfaces. The final ∆f and ∆D shifts are reported for molecular deposition in ethanol and buffer conditions, and the data are presented as the mean ± standard deviation, which were computed from *n* = 3 runs. The shaded regions correspond to the typical measurement values for a complete monolayer.

A summary of the final QCM-D measurement responses is presented for molecular deposition of DOCP, ODPA, and DPA in the different solvent systems (Figure 3). According to the streamlined protocols used here, DOCP lipid in ethanol and buffer systems could form rigidly attached monolayers on the TiO_2_ surface. On the other hand, ODPA in ethanol formed a more disordered SAM while ODPA in buffer did not attach to the surface. Moreover, DPA in ethanol did not attach to the surface, while DPA in buffer exhibited more aggregate-like adsorption. Collectively, these findings support that DOCP is able to form rigid monolayers using both ethanol and buffer deposition protocols, whereas ODPA forms a less rigid monolayer, and DPA forms a disordered adlayer and/or adsorbed aggregates in a narrower range of conditions. Compared to the saturated single-chain properties of ODPA and DPA, the versatile SAM functionalization options afforded by DOCP could be related to its two hydrocarbon chains that improve intermolecular packing and/or the presence of a double bond in each chain that could induce intramolecular steric hindrance to orient chain packing in a more upward direction. By comparison, for ODPA and DPA molecules, intermolecular interactions were less appreciable due to having just one hydrocarbon chain (and shorter chain length too in the case of DPA), and molecular tilting can occur in those cases due to balancing intermolecular interactions and the headgroup-substrate energy [49], which helps to explain the solvent-dependent deposition outcomes.

### 3.3. Hybrid Lipid Bilayer Formation

**Figure 4:**
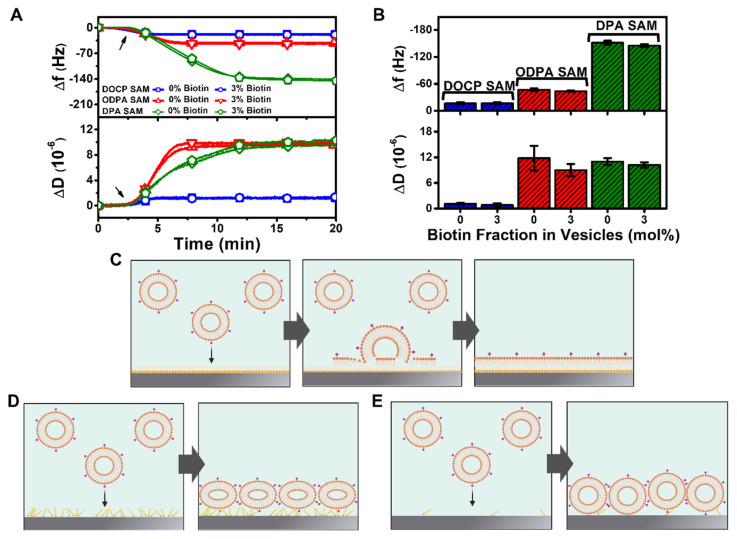
QCM-D tracking of vesicle adsorption and HLB formation on functionalized TiO_2_ surfaces. (**A**) Time-resolved ∆f and ∆D signals are reported for adsorption of vesicles containing 0 or 3 mol% biotinylated lipid onto TiO_2_ surfaces functionalized with DOCP, ODPA, or DPA molecules. The arrow indicates the start of vesicle addition. (**B**) Summary of final ∆f and ∆D shifts for vesicle adsorption corresponding to data in panel (**A**). The data are presented as the mean ± standard deviation from *n* = 3 runs. Schematic illustration of vesicle adsorption outcomes on TiO_2_ surfaces functionalized with (**C**) DOCP yielding HLB formation, (**D**) ODPA yielding intact vesicle adlayer formation (more deformed), and (**E**) DPA yielding intact vesicle adlayer formation (less deformed).

We proceeded to evaluate the interactions of ~80 nm diameter zwitterionic DOPC lipid vesicles containing 0 or 3 mol% biotinylated lipids with the TiO_2_ surfaces coated with DOCP, ODPA, and DPA in order to determine if HLB formation could be achieved (Figure 4). Note that the vesicle diameters were determined by dynamic light scattering (DLS) measurements. Based on the results described above, the DOCP- and ODPA-functionalized surfaces were fabricated using the ethanol deposition protocol, while the DPA-functionalized surface was fabricated using the buffer deposition protocol. Ideally, if the vesicles adsorb and spread on top of a hydrophobic SAM, a monolayer will form with an expected ∆f shift of around −13 Hz and ∆D shift of around or less than 1 × 10^−6^ [6]. Figure 4A presents the corresponding QCM-D vesicle adsorption kinetics onto the prefunctionalized TiO_2_ surfaces, while the final ∆f and ∆D shifts are also reported (relative to the initial buffer baseline for the prefunctionalized surfaces) (Figure 4B).

On the DOCP-functionalized surface, vesicles adsorbed monotonically, resulting in upper monolayer formation, and the one-step formation kinetics in this case are consistent with vesicle fusion driven by hydrophobic forces [6]. The interaction process resulted in HLB formation, and the corresponding ∆f and ∆D shifts for the vesicle addition were around −16.3 ± 2.3 Hz and 1.1 ± 0.2 × 10^−6^, respectively, for vesicles containing 0 mol% biotinylated lipids, which were within typical range for monolayer formation. Similar ∆f and ∆D shift values of around −16.4 ± 2.3 Hz and 0.8 ± 0.4 × 10^−^^6^, respectively, were recorded for vesicles containing 3 mol% biotinylated lipid, supporting that the presence of biotinylated lipids in the vesicles did not affect coating performance. In a separate experiment, we also tested vesicle adsorption onto DOCP-functionalized surfaces fabricated using the buffer protocol, and HLB formation was possible in that case as well (Figure S2).

On the ODPA-functionalized surface, vesicles also adsorbed monotonically but with appreciably larger QCM-D shifts, indicating that a monolayer did not form. For vesicles containing 0 mol% biotinylated lipids, the final ∆f and ∆D shifts were around −46.8 ± 2.8 Hz and 11.8 ± 2.9 × 10^−6^, respectively. Similar ∆f and ∆D shift values of −43.6 ± 1.1 Hz and 9.0 ± 1.4 × 10^−6^, respectively, were recorded for vesicles containing 3 mol% biotinylated lipid as well. These shifts were smaller than the typical ones for an intact vesicle layer [24,50], which suggests that there was a greater extent of vesicle deformation resulting from more attractive vesicle–surface interactions [51,52] compared to the intact vesicle layer case but not enough to cause vesicle rupture as in the case of HLB formation. Accordingly, a lower surface density of attached vesicles was present on the surface while there remained a high level of hydrodynamically coupled solvent imparting high viscoelastic character.

On the other hand, on the DPA-functionalized surface, vesicle adsorption also occurred with monotonic kinetics, but the corresponding ∆f and ∆D shifts were much larger and around −151.4 ± 4.3 Hz and 11.0 ± 0.8 × 10^−6^, respectively, for vesicles containing 0 mol% biotinylated lipid. There were similar ∆f and ∆D shift values of −144.9 ± 3.1 Hz and 10.2 ± 0.6 × 10^−6^, respectively, for vesicles containing 3 mol% biotinylated lipid. These measurement responses were consistent with typical values for intact vesicle adsorption on a bare TiO_2_ surface [50], which was consistent with a low amount of attached DPA on the TiO_2_ surface and hence DPA having minimal effect on vesicle adsorption properties.

In summary, vesicles adsorbed to form a rigid monolayer on the DOCP-functionalized surface that resulted in HLB assembly, while vesicles adsorbed but did not rupture on ODPA- or DPA-functionalized surfaces (Figure 4C–E). These findings support that the molecular features of the phosphate-functionalized amphiphiles deposited on the TiO_2_ surface had a strong influence on subsequent lipid self-assembly interactions and that DOCP lipids are particularly favorable to form a suitable SAM for HLB applications, at least using the streamlined deposition protocol devised here. We inferred from the cases of ODPA and DPA that surface hydrophobicity alone is insufficient to trigger vesicle rupture and subsequent HLB formation. This is consistent with a past report on HLB formation, in which case alkanethiol SAMs were tested, and a decrease in SAM ordering resulted in an increase in the polar part of the surface energy, which in turned lowered the adhesion energy and favored stabilization of lipid vesicles over vesicle fusion [53]. It has also been reported that, for alkanethiol SAMs, SAM chain length does not influence vesicle adsorption, but other factors, including SAM packing order, can affect sliding of the outer vesicle leaflet [54]. By contrast, the highly ordered DOCP SAM allows for a less polar surface free energy (i.e., more hydrophobic interactions between vesicles and the SAM surface) and hence facilitates vesicle rupture. Together, these findings demonstrate that HLB formation is possible on TiO_2_ surfaces, and the appropriate choice of the phosphate-functionalized molecular layer is a critical determinant of the fabrication outcome. Since DOCP lipids demonstrated high utility for HLB formation, we proceeded to focus subsequent biofunctionalization experiments on HLBs formed using DOCP lipid coatings obtained with ethanol and buffer deposition protocols.

### 3.4. Streptavidin Binding to HLB Platform

**Figure 5:**
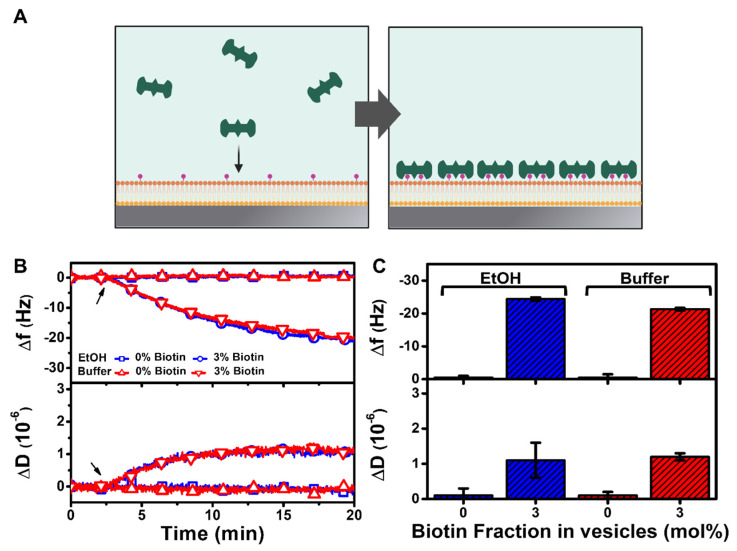
QCM-D tracking of streptavidin protein binding to HLB platform on a functionalized TiO_2_ surface. (**A**) Schematic illustration of streptavidin binding to HLB platform containing biotinylated lipids. The platforms were fabricated using DOCP-functionalized TiO_2_ surfaces that had been prepared using ethanol (EtOH) or buffer deposition protocols. (**B**) Time-resolved ∆f and ∆D signals are reported for streptavidin binding to HLB platforms containing 0 or 3 mol% biotinylated lipids. The arrow indicates the start of streptavidin addition. (**C**) Summary of final ∆f and ∆D shifts for streptavidin binding corresponding to data in panel (**A**). The data are presented as the mean ± standard deviation from *n* = 3 runs.

Streptavidin-biotin coupling is one of the most popular bioconjugation processes involved in biotechnology and biosensing applications, and hence we evaluated streptavidin protein binding to the HLB platforms fabricated on the TiO_2_ surface as a proof-of-concept experiment to verify binding selectivity (Figure 5A). We focused on the HLB platforms fabricated using DOCP-functionalized TiO_2_ surfaces and stabilized the platforms in buffer solution before adding 1 µM streptavidin in buffer solution with QCM-D measurement tracking. The HLB platforms containing 0 or 3 mol% biotinylated lipids were tested in order to evaluate resistance to nonspecific adsorption and selective binding of streptavidin, respectively.

When streptavidin was added to HLB platforms containing 3 mol% biotinylated lipids, there were large ∆f shifts of around −24 Hz along with moderate ∆D shifts of around 1 × 10^−6^, indicating rigid protein attachment (Figure 5B). In marked contrast, there were negligible ∆f and ∆D shifts for streptavidin binding to HLB platforms containing 0 mol% biotinylated lipid, verifying that protein attachment did not occur in the absence of biotinylated lipid receptors in the HLB platform. For the HLB platform containing 3 mol% biotinylated lipid that was prepared using the ethanol deposition protocol, the final ∆f and ∆D shifts for streptavidin addition were around −24.4 ± 0.5 Hz and 1.1 ± 0.5 × 10^−6^, respectively (Figure 5C). Similar measurement responses corresponding to final ∆f and ∆D shifts of around −21.3 ± 0.4 Hz and 1.2 ± 0.1 × 10^−6^, respectively, were also recorded for the HLB platform containing 3 mol% biotinylated lipid that was prepared using the buffer deposition protocol. These findings support that the HLB platform is suitable for selective attachment of streptavidin protein receptors and also prevents nonspecific adsorption of proteins in other cases due to the antifouling properties of the HLB coating.

### 3.5. Antibody-Antigen Detection

**Figure 6:**
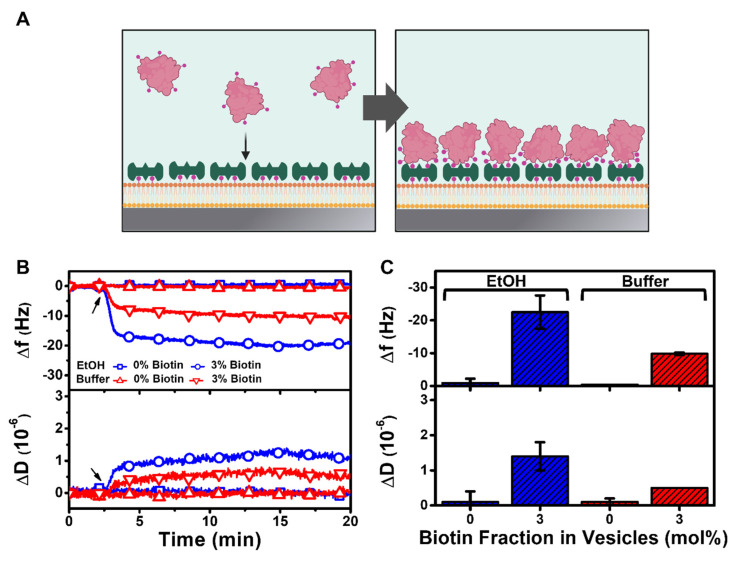
QCM-D tracking of biotinylated BSA protein attachment to a streptavidin-functionalized HLB platform. (**A**) Schematic illustration of biotinylated BSA attachment to a streptavidin-functionalized HLB platform. The platforms were fabricated using DOCP-functionalized TiO_2_ surfaces that had been prepared using ethanol (EtOH) or buffer deposition protocols. (**B**) Time-resolved ∆f and ∆D signals are reported for biotinylated BSA attachment to streptavidin-functionalized HLB platforms containing 0 or 3 mol% biotinylated lipids. The arrow indicates the start of biotinylated BSA addition. (**C**) Summary of final ∆f and ∆D shifts for biotinylated BSA attachment corresponding to data in panel (**A**). The data are presented as the mean ± standard deviation from *n* = 3 runs.

Next, we conducted antibody-antigen detection experiments using the HLB platform to demonstrate its potential utility for biosensing applications (Figure 6). While bovine serum albumin (BSA) protein itself does not usually adsorb to zwitterionic lipid bilayers, BSA can be functionalized with biotin moieties and then bind to streptavidin-functionalized lipid bilayers though noncovalent binding interactions mediated via biotin-streptavidin coupling (Figure 6A). Here, we tested the addition of 2 µM biotinylated BSA to streptavidin-functionalized HLB platforms that had been prepared using HLBs containing 3 mol% biotinylated lipids. Control experiments were prepared using HLBs that contained 0 mol% biotinylated lipid and followed the same subsequent protocol steps; in that case, no streptavidin was present due to the absence of biotinylated lipid in the HLB.

Based on these two platforms, the QCM-D measurements showed that there was significant biotinylated BSA attachment to the streptavidin-functionalized HLB, whereas there was no attachment to the control HLB platform (Figure 6B). For the biotinylated HLB prepared using the ethanol deposition protocol, the final ∆f and ∆D shifts due to biotinylated BSA attachment were around −22.5 ± 5.1 Hz and 1.4 ± 0.4 × 10^−6^, respectively (Figure 6C). Interestingly, for the biotinylated HLB prepared using the buffer deposition protocol, the final ∆f and ∆D shifts due to biotinylated BSA attachment were around −9.8 ± 0.4 Hz and ~0.5 × 10^−6^, respectively. This finding supports that the HLBs prepared using the different deposition protocols were both functional and enabled selective protein binding detection, while quantitative differences in the biotinylated BSA amount might relate to differences in the specific molecular properties of the two HLB platforms, e.g., amount and leaflet distribution of biotinylated lipids in the HLB and conformational properties of attached streptavidin molecules.

**Figure 7:**
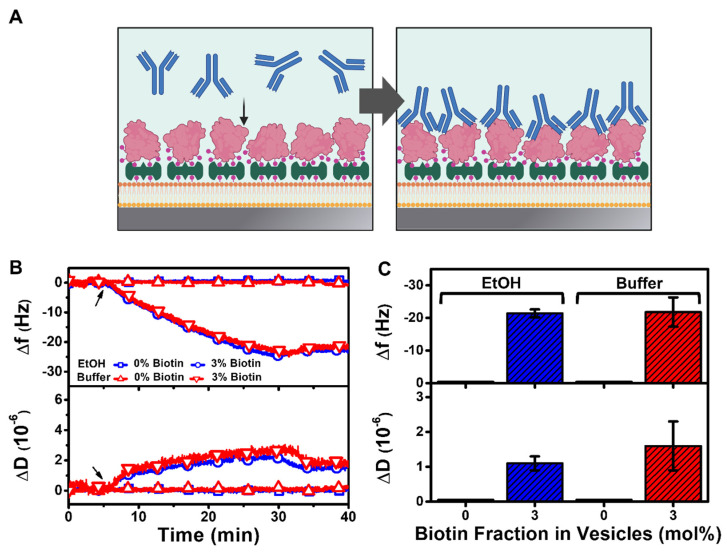
QCM-D tracking of BSA-specific antibody binding to membrane-associated BSA protein on HLB platform. (**A**) Schematic illustration of BSA-specific antibody binding to membrane-associated BSA protein on HLB platform. Note that the HLB platforms contained biotinylated lipid, to which subsequently added streptavidin protein binds, followed by biotinylated BSA that served as the antigen. The platforms were fabricated using DOCP-functionalized TiO_2_ surfaces that had been prepared using ethanol (EtOH) or buffer deposition protocols. (**B**) Time-resolved ∆f and ∆D signals are reported for BSA-specific antibody binding to membrane-associated BSA protein on HLB platforms containing 0 or 3 mol% biotinylated lipids. The arrow indicates the start of BSA-specific antibody addition. (**C**) Summary of final ∆f and ∆D shifts for BSA-specific antibody binding corresponding to data in panel (**A**). The data are presented as the mean ± standard deviation from *n* = 3 runs.

After biotinylated BSA attachment to the HLB platform, we proceeded to add 100 nM of BSA-specific antibody for a 20 min duration (Figure 7A). The QCM-D measurement responses showed that there was high antibody binding to the membrane-associated BSA protein on the biotinylated HLB platform, with maximum ∆f and ∆D shifts around −24 Hz and ~3 × 10^−6^, respectively (Figure 7B). On the other hand, no antibody binding was detected on the control HLB platform that lacked membrane-associated BSA, confirming that antibody binding was specific to the membrane-associated BSA antigen in this system. Of note, the corresponding ∆f and ∆D shifts due to antibody binding were around −21.4 ± 1.2 Hz and 1.1 ± 0.2 × 10^−6^, respectively, for the biotinylated HLB platform prepared using the ethanol deposition protocol (Figure 7C). Similar measurement responses with ∆f and ∆D shifts around −21.8 ± 4.5 Hz and 1.6 ± 0.7 × 10^−6^, respectively, were recorded for the biotinylated HLB platform prepared using the buffer deposition protocol. This finding supports that the HLB platform was capable of selective antibody detection and that the HLB platforms prepared using different solvent deposition protocols had similar performance for antibody detection. At the same time, it should be emphasized that standardization of HLB platform is important to control all stages of the fabrication process and resulting nanomaterial properties.

## 4. Conclusions

In this work, we investigated HLB formation using mono- (ODPA and DPA) and bi-tail phosphate-containing SAM molecules (DOCP) on TiO_2_ surfaces and devised a streamlined fabrication strategy to prepare HLBs that are useful for biosensing applications. We first deposited the SAM molecules in ethanol and aqueous buffer conditions to form an SAM and then added vesicles in cases where SAMs formed, in order to further evaluate the potential for HLB formation as well. The results showed that DOCP and ODPA formed SAMs when deposited in ethanol but only the DOCP SAM led to HLB formation upon vesicle adsorption. From a molecular viewpoint, the data support that only DOCP formed a rigid SAM when deposited in buffer and subsequent vesicle adsorption resulted in HLB formation, while ODPA did not adsorb but DPA did, albeit without forming a rigid SAM. Moreover, the DOCP-based HLBs allowed for the detection of antibody-antigen binding. Overall, these findings revealed that two-tail phosphate-containing SAM molecules, i.e., DOCP, were more suitable for HLB fabrication due to their molecular properties, which enabled the formation of a rigidly attached SAM on TiO_2_, and this capability demonstrated excellent potential for DOCP-supported HLBs to be applied in biosensing applications. 

## Data Availability

The data presented in this study are available upon reasonable request from the corresponding author.

## Supplementary Materials

The following supporting information can be downloaded at: www.mdpi.com/article/10.3390/nano12071153/s1, Figure S1: Magnified view for initial molecular deposition processes of DOCP lipid on TiO_2_ surface in ethanol and buffer. (A) Time-resolved QCM-D ∆f and ∆D signals are reported for DOCP deposition in ethanol and buffer solution. The arrows indicate the start of DOCP addition. (B) Summary of final ∆f and ∆D shifts for molecular deposition in ethanol and buffer conditions corresponding to data in panel (A). The data are presented as the mean ± standard deviation from n = 3 runs; Figure S2: QCM-D tracking of vesicle adsorption and HLB formation on DOCP-functionalized TiO_2_ surfaces in different solvent systems. (A) Time-resolved ∆f and ∆D signals are reported for adsorption of vesicles containing 0 or 3 mol% biotinylated lipid onto TiO_2_ surfaces functionalized with DOCP molecules in ethanol and buffer solution. The arrow indicates the start of vesicle addition. (B) Summary of final ∆f and ∆D shifts for vesicle adsorption corresponding to data in panel (A). The data are presented as the mean ± standard deviation from *n* = 3 runs.
