# Supplementary material for: Streamlined Fabrication of Hybrid Lipid Bilayer Membranes on Titanium Oxide Surfaces: A Comparison of One- and Two-Tail SAM Molecules
Source: Nanomaterials (Basel). 2022 Mar 30;12(7):1153. doi: 10.3390/nano12071153 (PMC9000636; doi:10.3390/nano12071153)
Supplement: Supplementary file 1 [file nanomaterials-12-01153-s001.zip › nanomaterials-1650827-supplementary.pdf]

*Supplementary Materials*

# Streamlined Fabrication of Hybrid Lipid Bilayer Membranes on Titanium Oxide Surfaces: A Comparison of One- and Two-Tail SAM Molecules

**Tun Naw Sut [1,†], Sue Woon Tan [1,†], Won-Yong Jeon [1], Bo Kyeong Yoon [2,*], Nam-Joon Cho [3,*] and Joshua A. Jackman [1,*]**

[1] School of Chemical Engineering and Translational Nanobioscience Research Center, Sungkyunkwan University, Suwon 16419, Korea; suttunnaw@skku.edu (T.N.S.); suewoon4695@gmail.com (S.W.T.); powerwy@skku.edu (W.-Y.J.)
[2] School of Healthcare and Biomedical Engineering, Chonnam National University, Yeosu 59626, Korea
[3] School of Materials Science and Engineering, Nanyang Technological University, 50 Nanyang Drive, Singapore 637553, Singapore
* Correspondence: bkyoon@jnu.ac.kr (B.K.Y.); njcho@ntu.edu.sg (N.-J.C.); jjackman@skku.edu (J.A.J.)
† These authors contributed equally to this work.

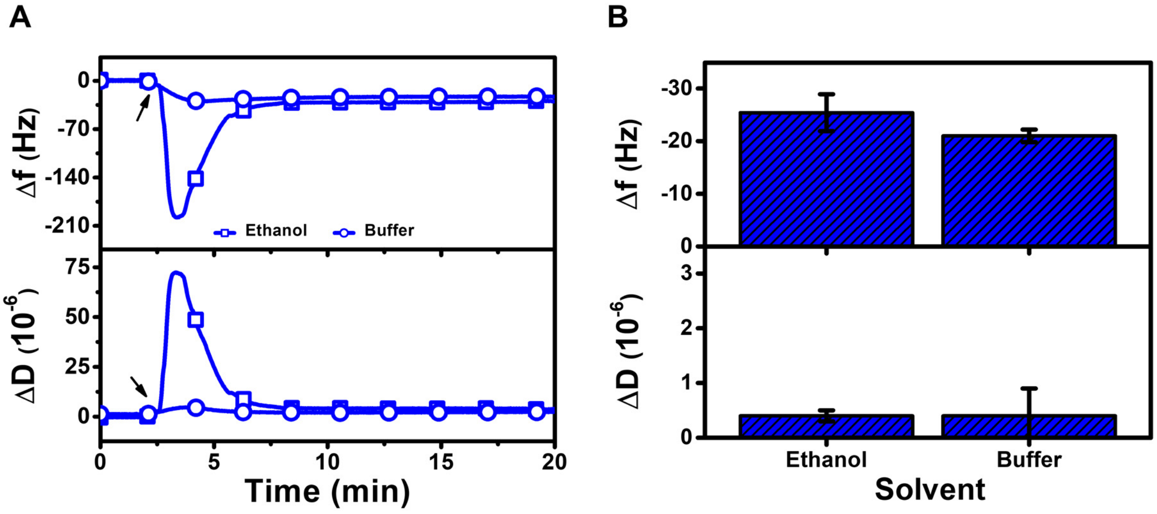


**Figure S1.** Magnified view for initial molecular deposition processes of DOCP lipid on $TiO_2$ surface in ethanol and buffer. (**A**) Time-resolved QCM-D Δf and ΔD signals are reported for DOCP deposition in ethanol and buffer solution. The arrows indicate the start of DOCP addition. (**B**) Summary of final Δf and ΔD shifts for molecular deposition in ethanol and buffer conditions corresponding to data in panel (**A**). The data are presented as the mean ± standard deviation from *n* = 3 runs.

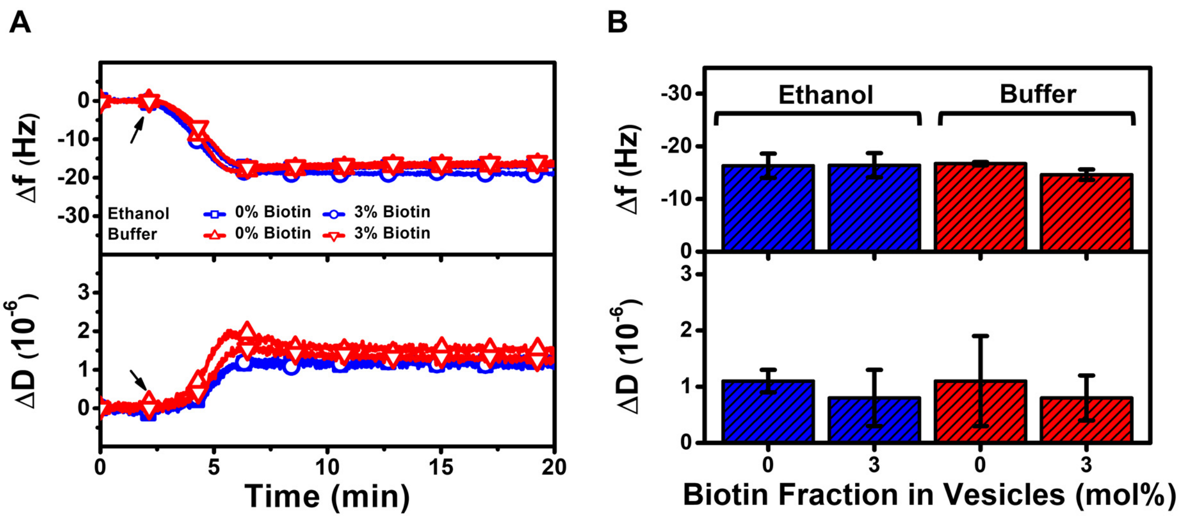


**Figure S2.** QCM-D tracking of vesicle adsorption and HLB formation on DOCP-functionalized $TiO_2$ surfaces in different solvent systems. (**A**) Time-resolved Δf and ΔD signals are reported for adsorption of vesicles containing 0 or 3 mol% biotinylated lipid onto $TiO_2$ surfaces functionalized with DOCP molecules in ethanol and buffer solution. The arrow indicates the start of vesicle addition. (**B**) Summary of final Δf and ΔD shifts for vesicle adsorption corresponding to data in panel (**A**). The data are presented as the mean ± standard deviation from $n$ = 3 runs.
